# If You Can't Measure It, You Can't Manage It

**DOI:** 10.1371/journal.pcbi.1003462

**Published:** 2014-03-20

**Authors:** Jean Peccoud

**Affiliations:** Virginia Bioinformatics Institute, Virginia Tech, Blacksburg, Virginia, United States of America; University of Bremen, Germany

## How the Lab Started

**Start of the lab**: 2006

**Size of the lab:** Currently ten—size has fluctuated between five and 20

**Research field:** Systems and synthetic biology

In 2005, I was working in the bioinformatics group of Pioneer Hi-Bred International, a subsidiary of DuPont. The company was downsizing its research and development effort. At the same time, synthetic biology was emerging as a new discipline, and I wanted to be part of this scientific adventure. Since it no longer seemed possible to work in this area at Pioneer, I looked at different career options like joining a synthetic biology startup or going back to academia. I had contacts at the Virginia Bioinformatics Institute (VBI), and they encouraged me to apply to an open position. They saw my experience in industry as a good fit with VBI entrepreneurial culture. I started my lab at VBI on May 1, 2006 with a gap in my publication record, no students, and an obligation to work in a field totally unrelated to what I had been doing for the previous four years while working at Pioneer.

## Scientific Mission of the Lab

We are building an integrated computational infrastructure to design, build, and test synthetic DNA molecules that meet user specifications [1]. This engineering challenge requires facing several difficult scientific questions. In particular, we are trying to model the relationship between structure and function in DNA sequences using formal languages [2]. One aspect of this broad question is how we should capture the context dependencies that affect the function of DNA sequences when placed in different genomic environments. We are using the regulatory network controlling the cell cycle in yeast as a model system to address these questions.

## Advice to a Beginning Researcher

I spent several years in industry between academic appointments. As a result, I see more commonalities than differences between these two worlds.

I share this experience with all of my students. Students need to understand that there are many meaningful jobs in industry, and these jobs are available at all levels. The students who spend some time in my lab are generally very successful academically, but few have any industry experience. They struggle to imagine any future outside of academia. Yet, only a small fraction of them will end up with faculty positions. So, I try to open their minds by explaining that academic jobs are not more desirable than industry jobs.

I strongly encourage junior PIs (principal investigators) who start their lab to look at it as a business whose primary customer is the government and whose primary function is to produce scientific articles. It needs a marketing strategy, a human resources strategy, a sales strategy, a financial strategy, and a production system that generates as many good-quality publications as possible, as inexpensively as possible. If a lab is seen as a small business, then the PI could be considered the CEO of that small business. He/she needs to make sure that all aspects of the business are functional; otherwise, there is a real possibility that the lab will go out of business and people will lose their jobs, including the PI if he/she is not tenured. Reading entry-level books on small business management [3] and innovation management [4] goes a long way to prevent this outcome.

*I strongly encourage junior PIs who start their lab to look at it as a business*

“You can't manage what you don't measure” is an old management adage that applies to academia. We know that bibliometric indicators are used to evaluate academic careers, but they do not say anything about the current performance of a research group. A junior faculty member may collect an impressive number of citations for work performed as a graduate student or during a postdoctoral training and yet do a lousy job as a PI. The PI needs instant metrics indicative of the conditions necessary to achieve long-term success.

## Time Management

A few years ago, I started wondering how much time I was spending on preparing research proposals. It felt like a lot, but I had no data. I needed good estimates to plan my proposal writing time. I started tracking the time spent on a few proposals. I was stunned by the results. It took me 80–100 hours per proposal. That's more than two weeks full time, assuming a 40-hour week. This number was interesting. Since there are no two weeks in the year I can dedicate entirely to writing a proposal, it meant that I needed to start earlier than I usually did, maybe a month or two before the deadline. Before I had data, I had the impression that resubmitting a proposal was faster than developing a new one. Wrong! The difference between writing a new proposal and recycling an old one was barely noticeable because the development of the research plan is only a fraction of the work involved in preparing a proposal. Furthermore, two closely related proposals still need distinct research plans that match the requirements of the funding agency and specific programs to which they are submitted.

*I started tracking time spent on proposals and was stunned by the results*

This data also led me to reassess the fraction of my time I should be spending on writing proposals. Statistics published by funding agencies show that the funding rate is rarely higher than 10% of the submitted proposals. In order to have one new grant per year, I should submit ten proposals, each requiring 100 hours to prepare. That's 1,000 hours, or half of the 2,000 work hours in a year. This calculation shed new light on my job. I had never thought of a PI job as being mostly about writing proposals. Up to that point, I thought of proposal writing as a necessary evil, akin to preparing my tax return. I had landed a sales job without even knowing it!

If tracking my time was very enlightening for me, chances were that it could benefit others as well. In my lab, we are now all tracking the use of our work time. We generate monthly indicators, such as the fraction of the time spent doing research on funded projects versus the time spent on nonresearch activities like writing proposals, taking classes, and performing administrative duties. The goal of the group is to spend 75% of our time on research. There are lots of differences between people. Postdoctoral research associates and staff are spending almost 90% of their time on research activities. Graduate students initially do very little research, as they spend a lot of time on class-related activities. As they get closer to graduation, they get close to spending 100% of their time on research. As for me, I can barely spend 33% of my time on research projects. We also keep track of the time spent on different research projects. This is important to make sure that the time people spend on different projects reflects the percentage of effort assigned to each grant.

## Number of Hours Worked

And yes, we do keep track of the total time spent working. It was difficult to make people understand that the purpose of tracking time was not to make them all work 100 hours per week but to develop an awareness of how they work. The rule is that we want to have 40 hours accounted for in every week for everyone. It should be work time or out-of-the-office time (vacation, medical leave). Everyone feels very busy. Yet, when looking at the total number of hours worked, people can be surprised to see that they have a hard time working 40 hours per week.

Travel is a good example for illustrating that feeling busy is not a good indicator of work time. On average, I make one or two trips per month. I have had some of my best writing time in airport lounges or on long international flights. However, while it may allow a few good hours of work time, travel also includes many hours where working is just not possible. On a recent trip back from Europe, I spent six hours at the gate waiting for a connection with a departure time that kept being pushed back in 30-minute increments while the ground crew was working on the plane. By then, I had been up for 24 hours, and I was so exhausted that I could no longer even watch a movie. Understanding where we spend our time is all the more important now that we can work from anywhere. Time worked no longer equates to time spent in the lab. When traveling, for instance, the time I can work is about 20 to 25% of the travel time.

## Fund-raising

Funding is another area where indicators can be useful. Initially, I was “chasing the money” by submitting proposals haphazardly in response to various calls of which I was aware. There were two problems with this approach. I did not understand well how to work with the different funding agencies, and I did not have the time to get the data and reputation necessary to get an award.

I now have a more strategic approach. I learned to work with two agencies (NSF [National Science Foundation] and NIH [National Institutes of Health]). I attended trainings to understand what they were looking for. I met with program directors. I served on review panels. And I wrote many proposals. Through practice, I learned to write them better and faster. I can now prepare most proposals in 50 hours or less.

Initially, I was trying to find something I could propose in response to a call. Now, I am committed to a few research projects that I need to get funded. New proposals are always somewhat related to ongoing projects but beyond the scope of what I can do with my current grants. I select the calls that fit my needs. If I think I can pitch my project to the call, then it is worth considering. It generally takes me three to five submissions over two to four years to get a project funded. Each set of reviews helps me revise and hopefully improve the proposal. However, it is also important to keep in mind that most programs receive many more high-quality proposals than they can fund. The selection of proposals that will receive funding among the pool of fundable proposals is somewhat reminiscent of a lottery. Submitting multiple high-quality proposals increases the odds of winning this game.

*I am committed to a few research projects that I need to get funded*

Another important consideration is the size of the funding available for the program. The size of the budget has little impact on the time it takes to prepare a proposal. Contrary to popular belief, it is not easier to get small grants than larger grants. If I am going to spend 50+ hours on a proposal, I may as well try to get $2M instead of $20,000. So, I ignore the smaller programs to focus on opportunities that can support large projects.

I developed a proposal preparation process that I follow religiously. It starts by identifying calls in which I am interested. I usually have their submission deadlines in my calendar six months ahead of time. This is necessary to avoid conflicts with other obligations, like travel. It is difficult to work on two proposals simultaneously, so I keep deadlines evenly spaced and try to submit one proposal a month.

*I developed a proposal preparation process that I follow religiously*

My proposal preparation process starts with circulating a one-page summary to potential collaborators. If they don't understand the summary, it is a waste of time to write the entire proposal. Then, before working on the narrative, I prepare the budget, as the story is in the budget! Budgetary constraints frame the narrative. If the program can only fund one graduate student and a small fraction of my time, the scope of the proposed research needs to reflect what can be accomplished with the resources of the program. Investigators who start by developing the narrative tend to write excessively ambitious research plans that cannot be supported by the program. Next, I make a list of all the documents needed to support the narrative. It takes time to get letters of commitment or quotes; it is therefore necessary to work on them as early as possible. I assemble all the supporting documents first because it is easy to sink an otherwise perfectly good proposal with sloppy supporting documents. For instance, a biography that has not been updated in years makes a very bad impression. Finally, I work on the narrative. I usually start by fleshing out the research plan and work on the justification, literature review, and presentation of preliminary data afterwards, since the goal of these sections is to support the research plan.

Opportunities to be a coinvestigator instead of the lead should not be rejected because of perceived loss of status. Looking at it pragmatically, it takes much less effort to join a proposal led by someone else than it takes to lead one. When looking at the budget divided by the number of hours spent on the proposal, joining proposals led by others becomes very attractive.

Determining the right number of collaborators is difficult. Adding collaborators often strengthens a proposal, but each one takes a share of a limited budget. I try to find a balance between increasing the odds of getting funding while ensuring that there is still enough money left for me to justify the effort. Another consideration is the collaborator commitment to the proposal; each collaborator is a potential point of failure during the preparation process because there is a possibility that they will not do their share of the work on time, which, in turn, may prevent me from submitting the proposal. This issue is particularly sensitive for collaborations between different institutions because they involve a lot of interactions between many stakeholders.

## Dissemination

I see social media as important components of scientific communication. We are present on Twitter, Facebook, Google+, and LinkedIn. We use Figshare to disseminate material that would not be accessible otherwise, such as posters, presentations, training material, datasets, and extended abstracts submitted to conferences. We also have a website that aggregates this information. Each channel is a way to communicate what we do to a broader audience. Just like a good marketing campaign can make a good product known to potential customers and poor marketing can lead to commercial failure, proper use of social media can increase the impact of scientific publications [5].

*I see social media as important components of scientific communication*

The emergence of article-level metrics measuring the impact of individual articles in social media helps fine-tune the communication strategy. They evolve quickly and seem to be good predictors of the number of citations [6], [7]. PLOS has been the first publisher to advocate the use of article-level metrics. Other services are now available to get similar data on virtually any scientific publication. I pay attention to these numbers and encourage my students to actively participate in social media to increase the visibility of their work.

## Motivation

People's motivations depend a lot on their personal and professional situations, and it is important for the PI to respect that diversity. What everyone in the group has in common is a need to be paid at the end of the month. I often remind people that we are all in the same boat here; either we are successful as a group or we will all be in trouble. I celebrate our successes and analyze our struggles openly and regularly. For instance, I send an email praising the first author when a publication is accepted. I take the group to lunch around May 1st to celebrate the group anniversary. This is an opportunity to ask people to reflect on the past year and the upcoming one, give my perspective, and allow people to ask questions they would not think to ask in other circumstances.

We have a continuous improvement committee composed of senior members of my lab. This group determines the processes used in all aspects of the lab operations. Members of this group compile monthly reports of performance indicators, and these reports are posted on a board for everyone to see. Having this committee in charge of setting processes and assessing their effectiveness gives people control over their work environment, which is very motivating. For example, the continuous improvement committee is responsible for specifying the processes to order laboratory supplies, record information in the laboratory information system, and organize and clean the laboratory.

*We have a continuous improvement committee composed of senior members of my lab*

In addition, I ask everyone in the lab to generate monthly bibliometric and time-usage reports with the goal of increasing their awareness of their own performance. They set their own standards based on their professional goals, but measurement leads to improvement no matter what their personal goals are.

## Useful Resources

**Toggl** (www.toggl.com): Time tracking software, free for a basic account.

**HootSuite** (www.hootsuite.com): Social media management tool, free basic account.

**Figshare** (www.figshare.com): Free repository to publish any research output, unlimited storage space but limits on the size of data sets.

**Altmetric** (www.altmetric.com): Article-level metrics.

**Peccoud Lab** (www.peccoud.org): Includes a blog on lab management.

**Figure:**
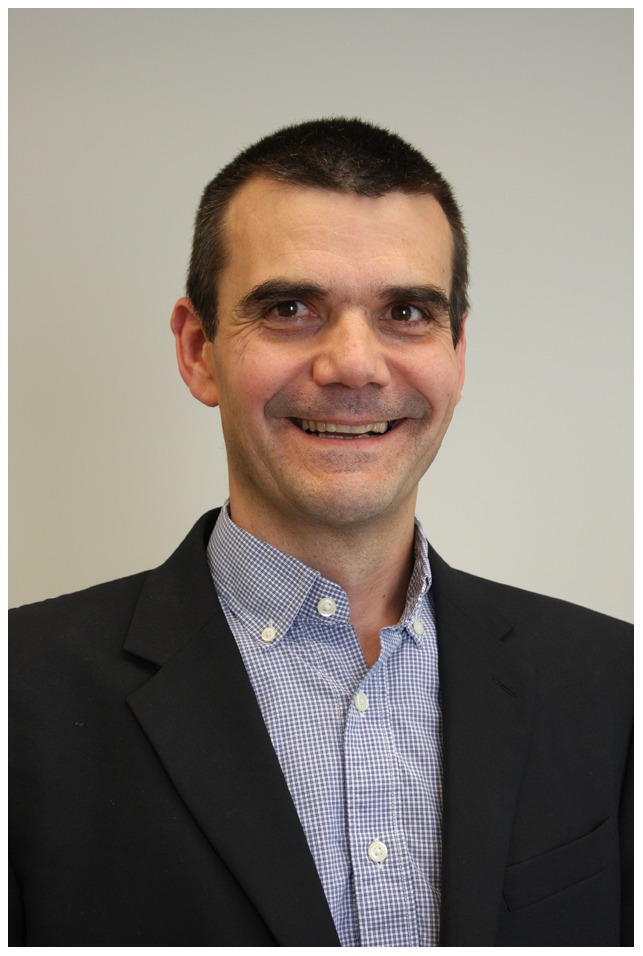
Image 1. Jean Peccoud; photo courtesy of Ivan Morozov, Virginia Tech.

## References

[1] Lux MW, Bramlett BW, Ball DA, Peccoud J (2012) Genetic design automation: Engineering fantasy or scientific renewal? Trends Biotechnol 30: 120–126. 22001068 10.1016/j.tibtech.2011.09.001 PMC3779889

[2] Cai Y, Lux MW, Adam L, Peccoud J (2009) Modeling structure-function relationships in synthetic DNA sequences using attribute grammars. PLOS Comput Biol 5: e1000529. 19816554 10.1371/journal.pcbi.1000529 PMC2748682

[3] Gerber ME (1995) The E-myth revisited: Why most small businesses don't work and what to do about it. New York (New York): HarperBusiness. xvi, 268 p.

[4] Ries E (2011) The lean startup: How today's entrepreneurs use continuous innovation to create radically successful businesses. New York: Crown Business. 320 p.

[5] Scott DM (2011) The new rules of marketing & PR: How to use social media, online video, mobile applications, blogs, news releases, and viral marketing to reach buyers directly. Hoboken (New Jersey): John Wiley & Sons. xxxii, 366 p.

[6] Priem J, Groth P, Taraborelli D (2012) The altmetrics collection. PLOS One 7: e48753. 23133655 10.1371/journal.pone.0048753 PMC3486795

[7] Neylon C, Wu S (2009) Article-level metrics and the evolution of scientific impact. PLOS Biol 7: e1000242. 19918558 10.1371/journal.pbio.1000242 PMC2768794

